# An application of the density standard and scaled–pixel–counting protocol to assess the radiodensity of equine incisor teeth affected by resorption and hypercementosis: preliminary advancement in dental radiography

**DOI:** 10.1186/s12917-023-03675-4

**Published:** 2023-08-09

**Authors:** Kamil Górski, Marta Borowska, Bernard Turek, Marek Pawlikowski, Krzysztof Jankowski, Andrzej Bereznowski, Izabela Polkowska, Małgorzata Domino

**Affiliations:** 1 Department of Large Animal Diseases and Clinic, Institute of Veterinary Medicine, Warsaw University of Life Sciences (WULS – SGGW), Nowoursynowska 100, 02-797 Warsaw, Poland; 2 Institute of Biomedical Engineering, Faculty of Mechanical Engineering, Białystok University of Technology, Wiejska 45C, 15-351 Bialystok, Poland; 3 Institute of Mechanics and Printing, Warsaw University of Technology, Narbutta 85, 02-524 Warsaw, Poland; 4 Division of Veterinary Epidemiology and Economics, Institute of Veterinary Medicine, Warsaw University of Life Sciences, Nowoursynowska 159C, 02-776 Warsaw, Poland; 5 Department and Clinic of Animal Surgery, Faculty of Veterinary Medicine, University of Life Sciences in Lublin, Głęboka 30, 20-950 Lublin, Poland

**Keywords:** Radiographs, Radiodensity, EOTRH, Dental care, Horse

## Abstract

**Background:**

Equine Odontoclastic Tooth Resorption and Hypercementosis (EOTRH) syndrome is a dental disease where the radiographic signs may be quantified using radiographic texture features. This study aimed to implement the scaled–pixel–counting protocol to quantify and compare the image structure of teeth and the density standard in order to improve the identification of the radiographic signs of tooth resorption and hypercementosis using the EOTRH syndrome model.

**Methods and results:**

A detailed examination of the oral cavity was performed in 80 horses and maxillary incisor teeth were evaluated radiographically, including an assessment of the density standard. On each of the radiographs, pixel brightness (PB) was extracted for each of the ten steps of the density standard (S1–S10). Then, each evaluated incisor tooth was assigned to one of 0–3 EOTRH grade–related groups and annotated using region of interest (ROI). For each ROI, the number of pixels (NP) from each range was calculated. The linear relation between an original X–ray beam attenuation and PB was confirmed for the density standard. The NP values increased with the number of steps of the density standard as well as with EOTRH degrees. Similar accuracy of the EOTRH grade differentiation was noted for data pairs EOTRH 0–3 and EOTRH 0–1, allowing for the differentiation of both late and early radiographic signs of EOTRH.

**Conclusion:**

The scaled–pixel–counting protocol based on the use of density standard has been successfully implemented for the differentiation of radiographic signs of EOTRH degrees.

## Background

The horse's ability to chew and grind food matter correctly is an important factor in the success of every nutritional, metabolic, and gastrointestinal case [1]. In this new era, in which the detailed dental examination is becoming a standard offering in the equine veterinary practice, the potential advancement in equine dentistry drives a change from lay–dentistry to clinical veterinary dentistry [2]. Recent developments in equine dentistry consist of advances in anatomical and physiological investigations [3, 4], development of modernized equine dental equipment [5], introduction of minimally invasive surgical techniques [6], and application of diagnostic imaging techniques translated from those used in human [5, 7, 8] and canine [9] dentistry.

In human dentistry, the assessment of bone and/or tooth quality is an important part of numerous dental manipulation protocols for example implant insertion [10] and accelerated orthodontic treatment [11]. The bone quality is best assessed by combining evaluation of bone mineral density and trabecular structure, which can be achieved radiographically using high–resolution imaging modalities such as high–resolution peripheral quantitative computed tomography (hr pQCT), multi–detector computed tomography (MDCT), or high resolution–magnetic resonance imaging (hr MRI) [12]. As most of the high–resolution imaging modalities have limitations in human clinical practice [10, 12], the use of alternative systems, such as cone–beam computed tomography (CBCT), have been considered [11, 12]. Therefore, in recent years, CBCT has been used in human dentistry as a diagnostic imaging modality to assess bone quality before implant surgery [13–15]. CBCT imaging modality, even though not high–resolution, delivers Hounsfield units (HU) reflecting bone quality [14–17].

In equine dentistry, CBCT and fan–beam computed tomography (FBCT) have already been used in a diagnostic imaging of equine head. Both CBCT and FBCT modalities were used for cadaver head scanning to detect dental and sinus abnormalities [8] and anatomical advancements [4, 18], respectively. Although CBCT and FBCT have a substantial agreement in detecting dental and sinus abnormalities in equine cadaver heads [8], one may observe that in the case of the living horse's head scanning FBCT was used as the preferred modality to detect signs of dental disease [19, 20], sinonasal cysts [21, 22], osteoma, and progressive ethmoid haematomas [19]. Some authors have compared and validated the accuracy of FBCT and radiographic imaging in detecting cheek [20, 23] or incisor teeth [24] disorders again, on both cadaver [23, 24] and living horse's heads [20]. Although computed tomography (CT) modality complement and overcome the limitations of two–dimensional radiographic images [25], standard radiography is widely used in equine practice for diagnoses and treatment purposes [26–28]. In equine dentistry, CT modality remains a costly imaging technique, which is restricted to universities or large clinical centers [29, 30] and contraindicated when deep sedation or general anesthesia is not performed [31]. Therefore, from the equine practitioner's point of view, standard radiography is recommended in the equine practice as a first choice method [28] for incisor [32] or cheek [33] teeth imaging.

Both CT modality and standard radiography represent techniques based on the detection of X–ray beam attenuation [34]. As in the case of CT, the bone or tooth quality assessment is based on a linear transformation of the original linear attenuation coefficient measurement into the HU scale [11], an analogous transformation is not available for radiographs. On the HU scale, the radiodensity at standard temperature and pressure is defined as ‑1000 HU for air, 0 HU for distilled water, 20–100 HU for soft tissue, up to 1000 HU for bone, and 2000 HU for dense bone or tooth [35]. However, one should note that HU values obtained from CT and returned without the use of specific scaled software such as the Slice Pick module and Bone Investigational Toolkit [36] are relative and do not represent absolute HU values [11]. Therefore, we hypothesize that radiographs in equine dental practice may be more specifically analyzed using a transformation model from standard radiographs to a numerical density scale similar to HU for CT scans.

Teeth demonstrate high radiodensity and are radiographically well–defined [37], thus some dental diseases with radiographic signs of radiodensity alteration can be used to verify the hypothesis. Noteworthy are hypo– and hypercementosis, in which decrease or increase radiopacity are easily detected, respectively, in the background of the high radiodense tooth structure [37, 38]. Hypocementosis of the infundibula of cheek teeth is a common abnormality of cemental development, which is visible radiographically in the form of decrease radiopacity. This cemental defect affects most commonly the apical region of the infundibulum as a result of the reduction in the vascular supply to the mesial infundibulum [39]. Hypercementosis of incisor and canine teeth is also a common abnormality, however, appears generally in horses over 14 years of age, and is visible radiographically in the form of increase radiopacity. This cemental accumulation occurs alone or together with tooth resorption in the case of Equine Odontoclastic Tooth Resorption and Hypercementosis (EOTRH) syndrome [40], which may involve the whole tooth structure. Hypercementosis mainly appears as a result of cement accumulation forming bulbous enlargements on the reserve crown and/or apex of the tooth [41], whereas resorption affects the enamel, cementum, dentine, and pulp cavity [40]. So far various aetiologies of EOTRH syndrome have been proposed, however, as yet none have been substantiated [2, 32]. Comparing these two diseases, EOTRH syndrome is easier to evaluate using standard radiographic technique due to the lower superimposition of soft tissue related to the favorable rostral position of the incisor than cheek teeth [42]. Infundibular hypocementosis of cheek teeth is more difficult to evaluate using standard radiographic technique due to the higher superimposition of soft tissues and the contrast reduction caused by the peripheral and infundibular enamel [43]. Therefore, standard radiographs of incisor teeth affected by EOTRH syndrome were selected as the first equine model suitable for the testing of the relative tooth quality evaluation using the scaled–pixel–counting protocol.

This study aimed to implement the scaled–pixel–counting protocol to quantify and compare the image structure of teeth and density standard in order to improve the identification of the radiographic signs of tooth resorption and hypercementosis using the EOTRH syndrome model.

## Materials

### Animals and study design

The study was conducted on 80 privately owned horses (age mean ± standard deviation (SD): 16.9 ± 7.0; 37 geldings, 43 mares; 30 Polish Halfbred horses, 13 Arabian horses, 10 Schlesisches Warmblood horses, 8 Wielkopolska breed horses, 7 Dutch Warmblood horses, 5 Thoroughbred horses, 4 Polish draft horses, and 3 Malopolska breed horses) between July 2021 and December 2021. The health status of the horses was inspected according to veterinary standards including a basic clinical examination [44] a detailed examination of the oral cavity [45], and an X–ray examination of the maxillary incisor teeth using the intra–oral dorso–ventral projection [32].

The X–ray images of the horses' incisor teeth and density standard were taken simultaneously, and the study was performed according to the following five–step protocol:

(i) classification of each incisor tooth of every horse to one of the four grade–related EOTRH groups (0–3); (ii) annotation of the regions of interest (ROIs) of the incisor teeth and the density standard; (iii) implementation of the scaled–pixel–counting protocol to quantify and compare the image structure of teeth and density standard; (iv) comparison of the quantification results; (v) assessment of the accuracy of identification of grade–related EOTRH groups (0–3) based on the quantification results.

### Classification of horses' incisor teeth

A basic clinical examination aimed to investigate the internal temperature, heart rate, respiratory rate, mucous membranes, capillary refill time, and lymph nodes in order to qualify the horses for the sedation procedure. No clinical contraindication to the sedation procedure were found in any of the examined horses. A basic clinical examination was conducted following standard protocol [44].

The sedation procedure aimed to prepare the horses for a detailed examination of the oral cavity. Each horse received a dose of detomidine hydrochloride (0.01 mg/kg bwt i.v. of Domosedan; Orion Corporation, Espoo, Finland), or xylazine hydrochloride (0.4 mg/kg bwt i.v. of Xylapan; Vetoquinol Biowet Sp. z o.o., Gorzów Wielkopolski, Poland), or a combination of both. In some cases, horses received a dose of butorphanol tartrate (0.01 mg/kg bwt i.v. of Torbugesic; Zoetis Polska Sp. z o.o., Warsaw, Poland).

A detailed examination of the oral cavity aimed to collect the clinical signs of dental diseases, concerning the condition of teeth, interdental spaces, gums, and mucosa of the cheeks and tongue. This examination was conducted by visual examination as well as manual and using periodontal probe palpation after the mouth opening by a Haussmann's mouth speculum and the oral cavity flushing by a 400 mL syringe. Each dental tool was used in a manner that ensured the safety of the horse and veterinarian during examination. The oral cavity was flushed to remove any food which remained on, around, and between the teeth, as well as to evaluate the interdental spaces, respectively. A detailed examination of the oral cavity was conducted following standard protocol [45] and was documented using an equine dental chart [46].

**Fig. 1:**
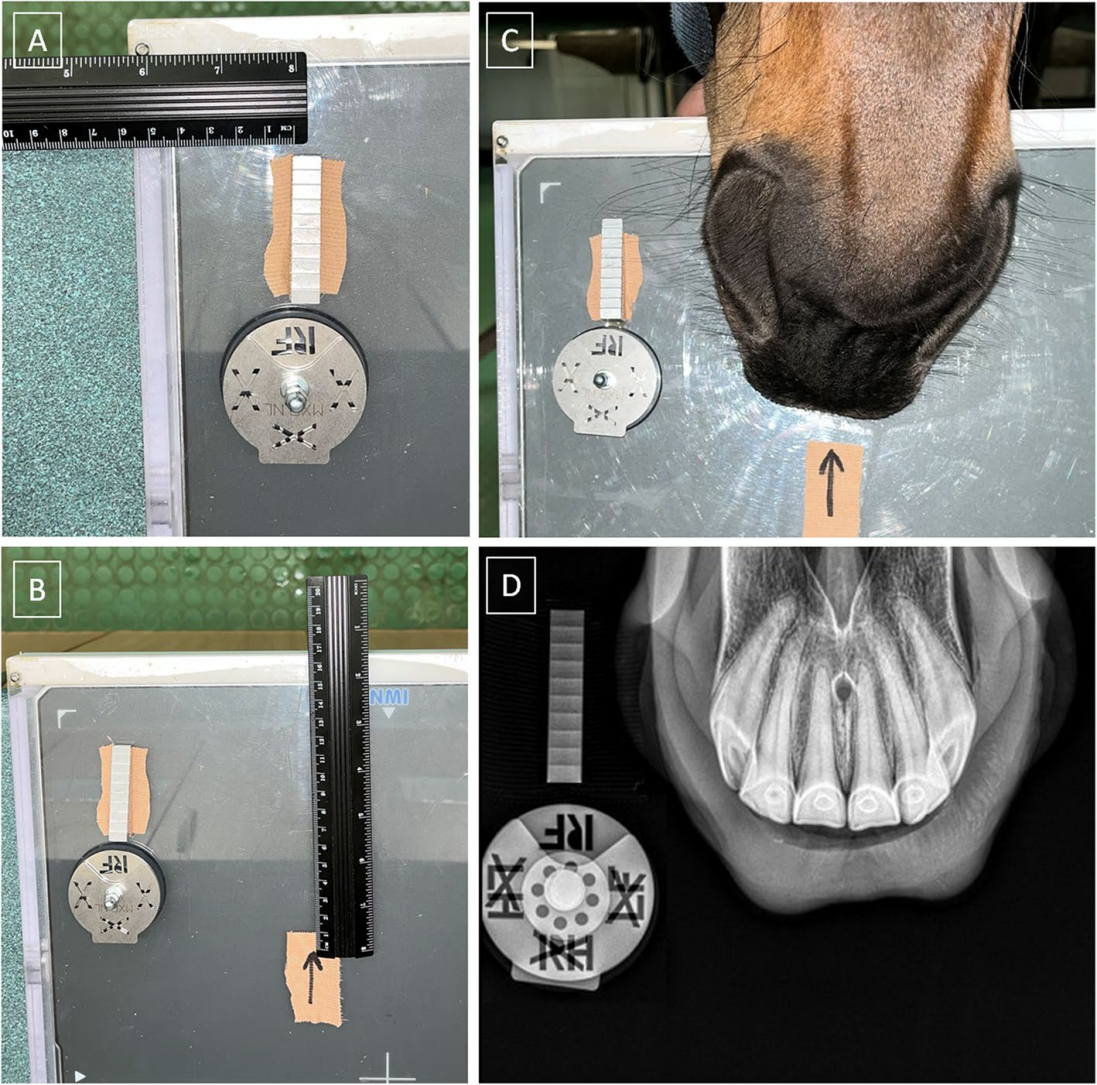
The positioning of density standard (**A**), specific place of the cassette (**B**), and horse's nostrils (**C**) while obtaining the intra–oral dorso–ventral projection of the incisor teeth, density standard, and directional indicator (**D**)

An X–ray examination of the maxillary incisor teeth aimed to collect the radiographic signs of EOTRH syndrome including shape, contour, radiodensity, and delineation of the periodontal space [32]. This examination was conducted using the intra–oral dorso–ventral projection by inserting the protected radiographic cassette into the horse's oral cavity [47] as well as the guidelines of the bisecting angle technique [48]. A density standard patch was attached to the upper right corner of the radiographic cassette. Such positioning allowed to minimize the absorption of ionizing radiation, and thus false results. A density standard was positioned perpendicular to the surface of the cassette, 4 cm from the top and 4 cm from the right edge of the cassette so that the long axis of the density standard was parallel to the long axis and the thick end was caudally of the cassette (Fig. 1A). The cassette was positioned in the horse's open mouth, always the same distance from to the density standard, 15 cm from the top and 15 cm from the right edge of the cassette (Fig. 1B). The cassette was positioned so that the center of the horse's nostrils was in the specific place of the cassette, which was marked with a patch containing an arrow (Fig. 1C). The examination was conducted using an X–ray tube (Orange 9020HF, Ecoray Co., Seoul, Korea), a radiographic cassette (Saturn 8000, Vievorks Co., Seoul, Korea), and a portable computer (HP Inc UK Ltd, Reading, UK). The X–ray tube settings were 2.5 mAs and 65 kV, and the distance between the X–ray tube and radiographic cassette was 80 cm. The radiographs were acquired as.jpg files and processed using the DxWorks software (Vievorks Co., Ltd., Seoul, Korea). An X–ray examination was conducted following standard protocol [32], so that the incisor teeth, density standard, and directional indicator were visible on the radiograph (Fig. 1D).

**Fig. 2:**
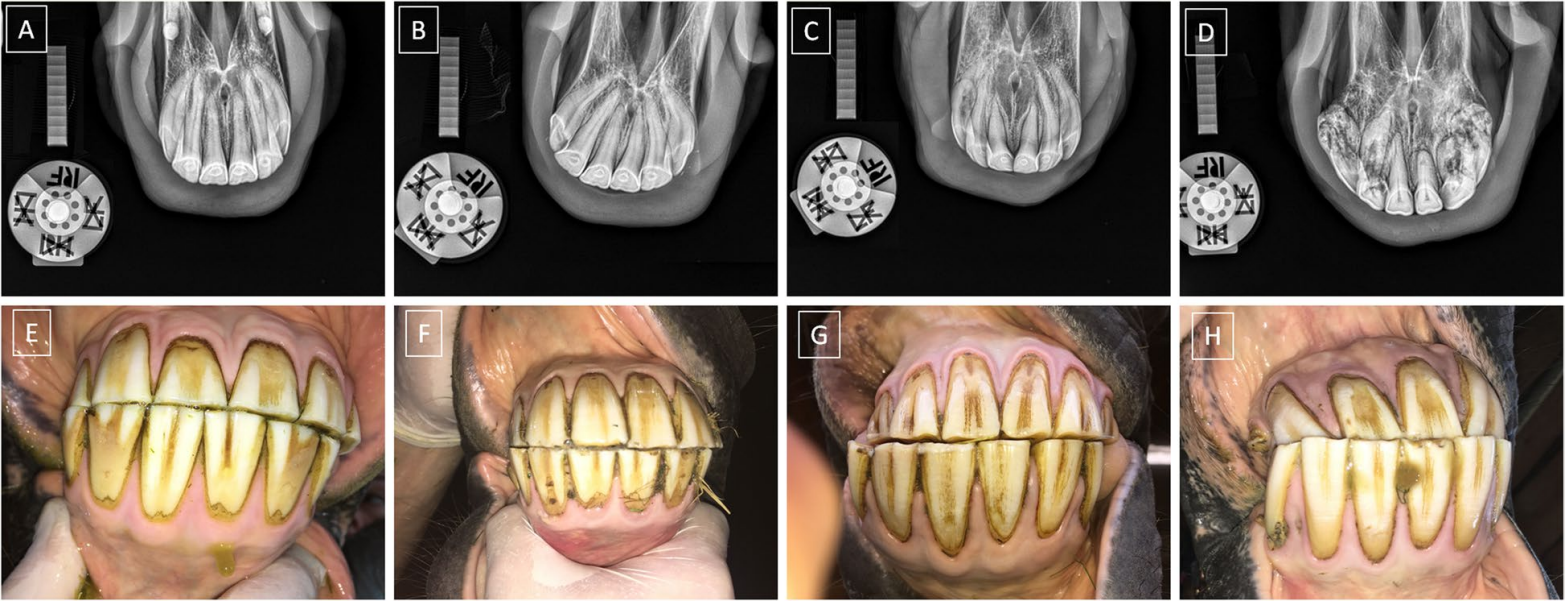
Example of radiographic (**A**–**D**) and clinical (**E**–**F**) images of the incisor teeth classified to grade-related Equine Odontoclastic Tooth Resorption and Hypercementosis (EOTRH) group 0 (**A**, **E**), group 1 (**B**, **F**), group 2 (**C**, **G**) and group 3 (**D**, **H**)

The horses' incisor teeth, numbered according to the modified Triadan system [49], were classified using the radiographic classification system introduced by Hüls et al. [50] and modified by Rehl et al. [32]. Each incisor tooth, numbered as 101, 102, 103, 201, 202, or 203, was evaluated and annotated to one of the four grade–related EOTRH groups (0–3). The inclusion criterion for group 0 was no radiographic signs of EOTRH syndrome (Fig. 2A, E). The inclusion criteria for group 1 were preserved tooth shape or slightly blunted root tip as well as irregular or rough tooth surface (Fig. 2B, F). The inclusion criteria for group 2 were largely preserved tooth shape or that the intra–alveolar tooth part was not wider than the clinical crown, or obviously blunted root tip as well as irregular or rough tooth surface (Fig. 2C, G). The inclusion criteria for group 3 were loss of tooth shape or a wider intra–alveolar tooth part compared to the clinical crown, as well as obviously irregular or rough tooth surface (Fig. 2D, H). The exclusion criterion was presence of clinical and radiographic signs of diseases of the incisor teeth, including: supernumerary teeth, loose teeth, fractures, caries, and calculus [46].

### Characteristic of density standard

**Fig. 3:**
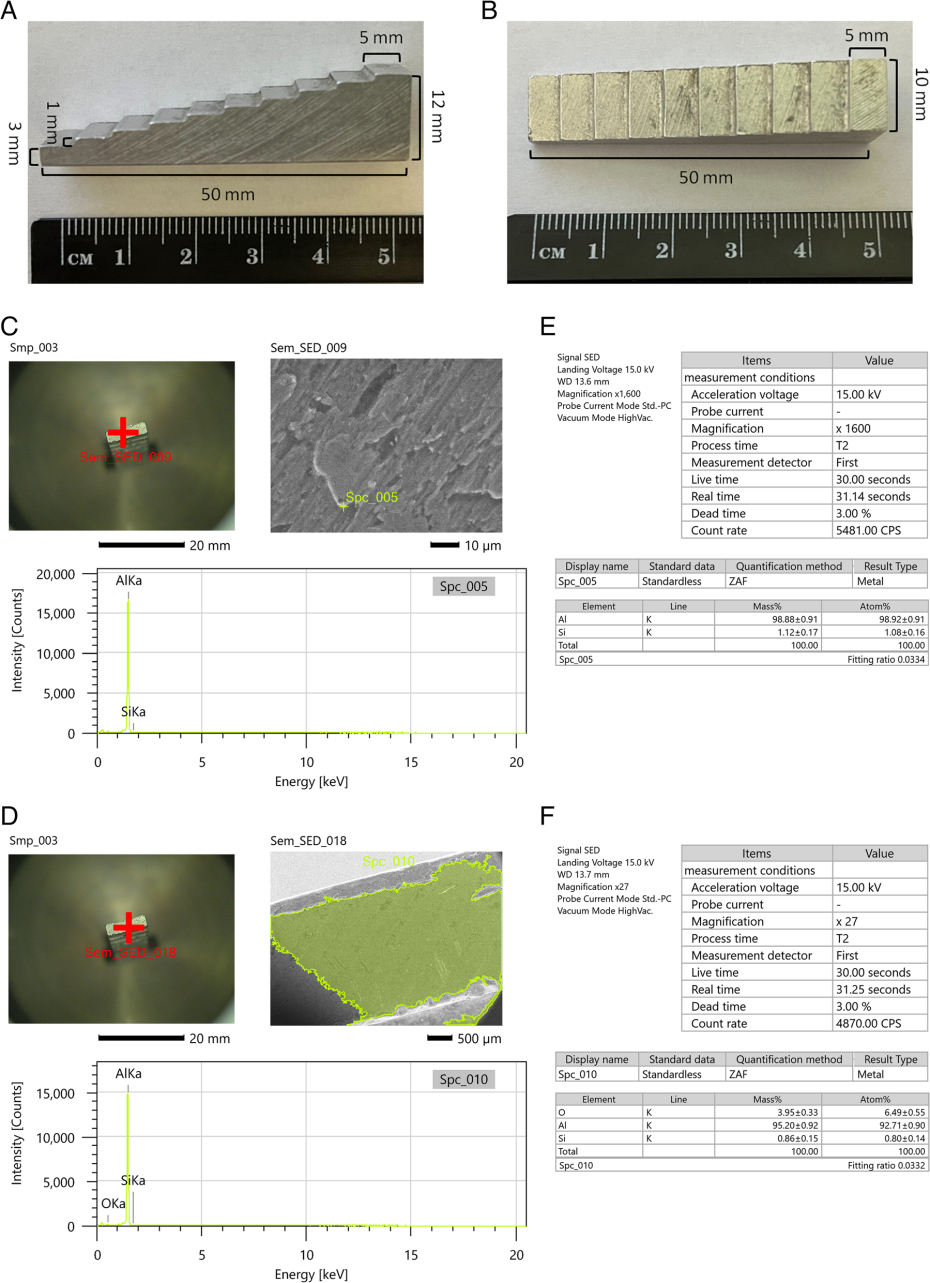
The lateral projection (**A**) and perpendicular to the base projection (**B**) of density standard with the marked dimensions, as well as the point (**C**) and surface (**D**) composition of density standard sample evaluated under a scanning electron microscope (SEM) and corresponding to Mass% and Atom% (E, F)

Density standard, with a volume of 3545.93 mm^3^ and dimensions: 55 mm length of the basis, 12 mm high in the highest place, 3 mm high in the lowest place, and 10 mm width, was used in this study (Fig. 3A). Density standard had the shape of an irregular cuboid with 10 steps (S1–S10) decreasing the height of the cuboid in the projection perpendicular to the base. Each step was 5 mm long, 1 mm high, and 10 mm wide, with the exception of the lowest step, which was 3 mm high (Fig. 3B). Density standard, with a mass of 9.39 g and a density of 2.65 g/cm^3^, consisted of aluminum (Al) with a point intensity of 17 000 counts at 1.52 keV energy (Fig. 3C) and a surface intensity of 15 000 at 1.52 keV energy (Fig. 3D) measured under the scanning electron microscope (SEM) (JCM–7000 NeoScope™ Benchtop SEM, JEOL, Tokyo, Japan), which corresponded to 95.20–98,88 Mass% and 92.71–98.92 Atom% of Al (Fig. 3E–F), respectively.

**Fig. 4:**
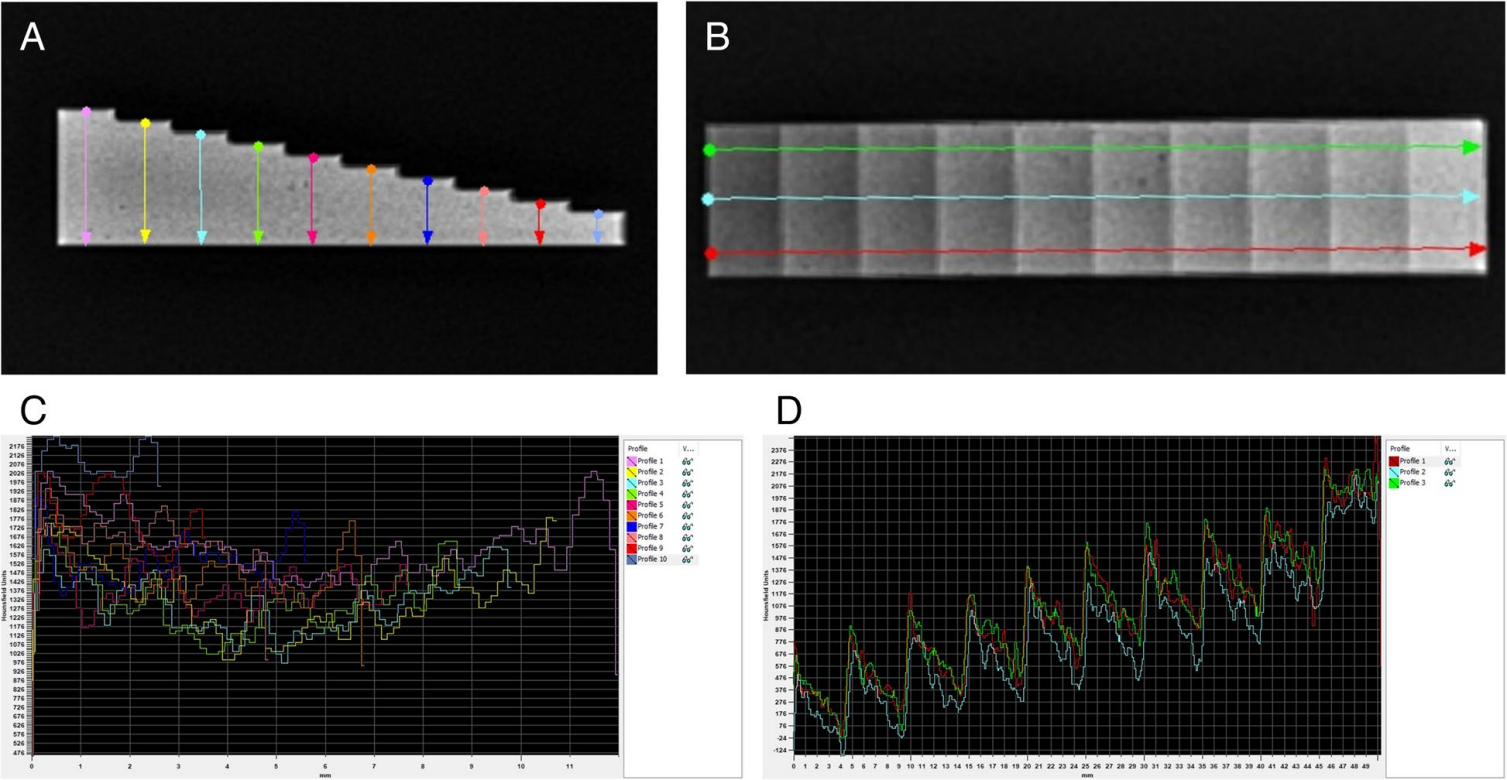
The lateral projection (**A**) and top–bottom projection (**B**) of density standard with the marked measuring lines, as well as the values of ten vertical (**C**) and three horizontal (**D**) attenuations of the X–ray beam passing through the density standard evaluated by Materialises interactive medical image control system (MIMICS) and corresponding to Hounsfield unit (HU) (**E**, **F**)

The reference attenuation of the X–ray beam passing through the density standard was analysed by Materialises interactive medical image control system (MIMICS) software (Materialise HQ, Leuven, Belgium) on two referential X–ray images obtained using 2.5 mAs and 65 kV X–ray tube settings with a 80 cm distance between X–ray tube and radiographic cassette (X–ray tube, Orange 9020HF, Ecoray Co., Seoul, Korea; radiographic cassette, Saturn 8000, Vievorks Co., Seoul, Korea; portable computer, HP Inc UK Ltd, Reading, UK). The attenuation of the X–ray beam passing the density standard were presented as HU for two projections perpendicular to each other – lateral projection (Fig. 4A) and top–bottom projection (Fig. 4B). On the lateral projection, ten measuring lines corresponding to the location of the middle of S1–S10 were marked by different colors, and the HU values were displayed on the plot of HU versus distance (Fig. 4A). On the top–bottom projection, three measuring lines corresponding to the lateral, middle, and medial longitudinal sections of density standard were marked by different colors, and the HU values were displayed on the plot of HU versus distance (Fig. 4B). The values of HU measured for each of S1–S10 of density standard were summarized in Table 1 in the results section as a mean ± SD from all evaluated measuring lines.

**Table 1:** The values (mean ± standard deviation (SD)) of raw Hounsfield unit (HU) and normalized Hounsfield unit (nHU) as well as raw pixel brightness (PB) and normalized pixel brightness (nPB) measured for ten steps of density standard (S1–S10)

	S1	S2	S3	S4	S5	S6	S7	S8	S9	S10
HU mean	1009	1212	1407	1600	1804	2011	2204	2400	2607	2803
± SD	163	111	98	134	112	99	107	133	129	147
nHU	0	0.11	0.22	0.33	0.44	0.56	0.67	0.78	0.89	1.00
PB mean	83.0	93.5	101.1	108.5	115.2	121.3	127.7	133.7	143.1	168.6
± SD	10.5	10.5	11.3	12.0	12.6	12.9	13.8	14.5	16.7	17.6
nPB	0	0.07	0.17	0.21	0.26	0.29	0.35	0.42	0.53	1.00

### Annotation of ROIs

**Fig. 5:**
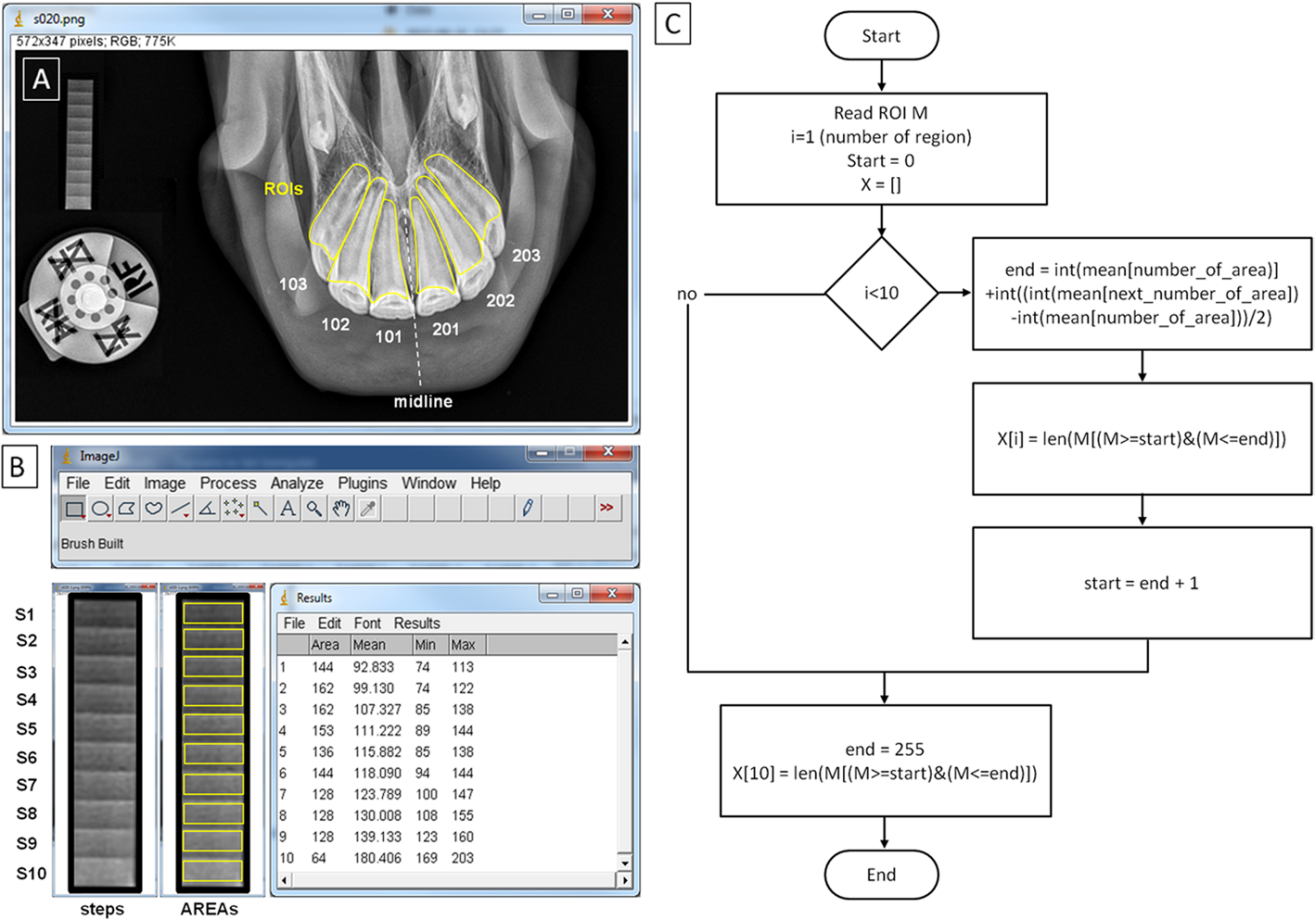
Regions of interest (ROIs) annotated on incisor teeth (**A**) and regions of interest annotated on ten steps of density standard (S1-S10) (AREAs) (**B**), and the algorithm to calculate the absorption degree ranges (**C**). The algorithm counts each ROI on the incisor teeth and the number of pixels from each range (S1–S10) representing the degree of X–ray beam attenuation

On each incisor tooth, the polymorphic ROI was manually annotated using the ImageJ software (version 1.46r, Wayne Rasband, Bethesda, MD, USA). Each ROI was individually fitted to the separate tooth (101, 102, 103, 201, 202, or 203) so that the ROI covered the largest possible area of the tooth crown and root (Fig. 5A).

On each radiograph, ten rectangular regions of interest representing S1–S10 (AREAs) were manually annotated using the ImageJ software (version 1.46r, Wayne Rasband, Bethesda, MD, USA) (Fig. 5B).

### Implementation of the scaled–pixel–counting protocol

The AREAs represented ten steps of density standard with various degrees of X–ray beam attenuation. Each AREAs returned the values of PB < 0; 255 > and determined the ranges of PB change in each AREAs according to the following formula:

$$<start\left[k\right],end\left[k\right]>=\langle end\left[k-1\right],\frac1{M\cdot N}\sum_{i=0}^{M-1}\sum_{j=0}^{N-1}AREA\left[k\right]\left[i,j\right]+\left(\frac1{M\cdot N}\sum_{i=0}^{M-1}\sum_{j=0}^{N-1}AREA\left[k+1\right]\left[i,j\right]-\frac1{M\cdot N}\sum_{i=0}^{M-1}\sum_{j=0}^{N-1}AREA\left[k\right]\left[i,j\right]\right)/2\rangle$$

where $$start\lbrack0\rbrack=0$$ and $$end\lbrack10\rbrack=255$$.

The values of PB measured for each AREAs of S1–S10 of density standard were summarized in Table 1 in the result section as a mean ± SD from all evaluated radiographs. Areas that attenuated a small amount of X–ray beam (representing soft tissue and tooth resorption) were dark, while areas that attenuated a large amount of X–ray beam (representing tooth and cement accumulation representing the areas of hypercementosis with the increased radiodensity compared to the normal lucency of the pulp canal) were bright. Thus, on the pixel brightness (PB) scale, the equivalent of radiodensity was visible in the form of different gray levels represented as 90 for soft tissue and tooth resorption, 140 for a normal tooth, and 190 for tooth cement accumulation.

These equivalents were counted for each ROI, representing each incisor tooth, as NP from each range (< PB S1; PB S10 >). In this way, the degree of X–ray beam attenuation for each incisor tooth was quantified in the form of the number of pixels (NP) data set of ten values (< NP1; NP10 >). The algorithm (Fig. 5C) was implemented in Python language.

### Statistical analysis

The S1–S10 data series of mean HU and mean PB were tested independently for univariate marginal distributions using the Shapiro–Wilk normality test. Since both data series were Gaussian distributed, the Pearson correlation coefficient (r) was calculated for raw data. The value of r reflected the consistency when the *p* < 0.05. Then, the normalizing of mean HU (nHU) and normalizing of mean PB (nPB) data series to the < 0,1 > range was performed. For nHU and nPB, the linear regressions were calculated. On the regression plot, regression equations for nHU and nPB were displayed and the slopes of both nHU and nPB were significantly non–zero (*p* < 0.0001). Both equations were supported with the measurement of the difference of linearity. For no significant difference between the slopes (*p* > 0.05), a single slope was calculated, and the intercepts were compared.

The whole NP data set, where each tooth of each horse represented one realization, was divided into four EOTRH grade–related groups, thus four EOTRH grade–labeled data series (EOTRH 0, EOTRH 1, EOTRH 2, EOTRH 3) were extracted. Each extracted EOTRH grade–related data series contained ten S–labeled data series (S1, S2, S3, S4, S5, S6, S7, S8, S9, S10). These forty data series were tested independently for univariate distributions using the Shapiro–Wilk normality test.

S–labelled data series were then compared between steps of density standard (S1–S10) using the Kruskal–Wallis test, followed by the Dunn’s multiple comparisons test. For each data set, at least one data series was non–Gaussian distributed. The alpha value was established as α = 0.05. Data were compared and displayed for each EOTRH group separately. The NP values were presented on plots with bars using mean + SD, where lower case letters indicated differences between steps.

EOTRH grade–labelled data series were then compared between EOTRH grades (EOTRH 0 – EOTRH 3) using the Kruskal–Wallis test, followed by the Dunn’s multiple comparisons test. For each data set, at least one data series was non–Gaussian distributed. The alpha value was established as α = 0.05. Data were compared and displayed for each step (S1–S10) separately. The NP values were presented on plots with bars using mean + SD, where lower case letters indicated differences between steps. On the respective plot, when an NP value was found to significantly increase with the EOTRH grade, the colored lines were additionally marked. An orange line was marked when the NP value increased between EOTRH 0 and 3, a red line was marked when the NP value increased between EOTRH 1 and 3, a blue line was marked when the NP value increased between EOTRH 0 and 1, and a green line was marked when the NP value increased between EOTRH 2 and 3.

Each step significantly increased with the EOTRH grades, and the accuracy of the differentiation of selected EOTRH grades was calculated using two thresholds (mean and |mean – SD|). EOTRH grade selection was marked on respective figures by the colored lines described above, thus the accuracy of differentiation of four EOTRH grade pairs, EOTRH 0 vs. 3, EOTRH 1 vs. 3, EOTRH 0 vs. 1, and EOTRH 2 vs. 3, was estimated. The incisor tooth was annotated with a lower EOTRH grade in a pair when the individual measured value was below the threshold and annotated as higher EOTRH grade in a pair when above the threshold. The standard formulae [51] were used to calculate the sensitivity (Se), specificity (Sp), positive predictive value (PPV), and, negative predictive value (NPV) in the < 0.1, 1.0 > range.

All statistical analysis was performed using Graph Pad Prism 6 software (GraphPad Software Inc., Avenida De La Playa La Jolla, CA, USA).

## Results

For density standard, mean values of HU ranged from 1009 to 2803 whereas mean values of PB ranged from 83.0 to 168.6 (Table 1). Both data series gradually increased with the thickness of the ten steps of the density standard from S1 to S10 and the Pearson correlation coefficient indicated statistically significant strong positive correlation between mean values of HU and PB (r = 0.978; *p* < 0.0001).

**Fig. 6:**
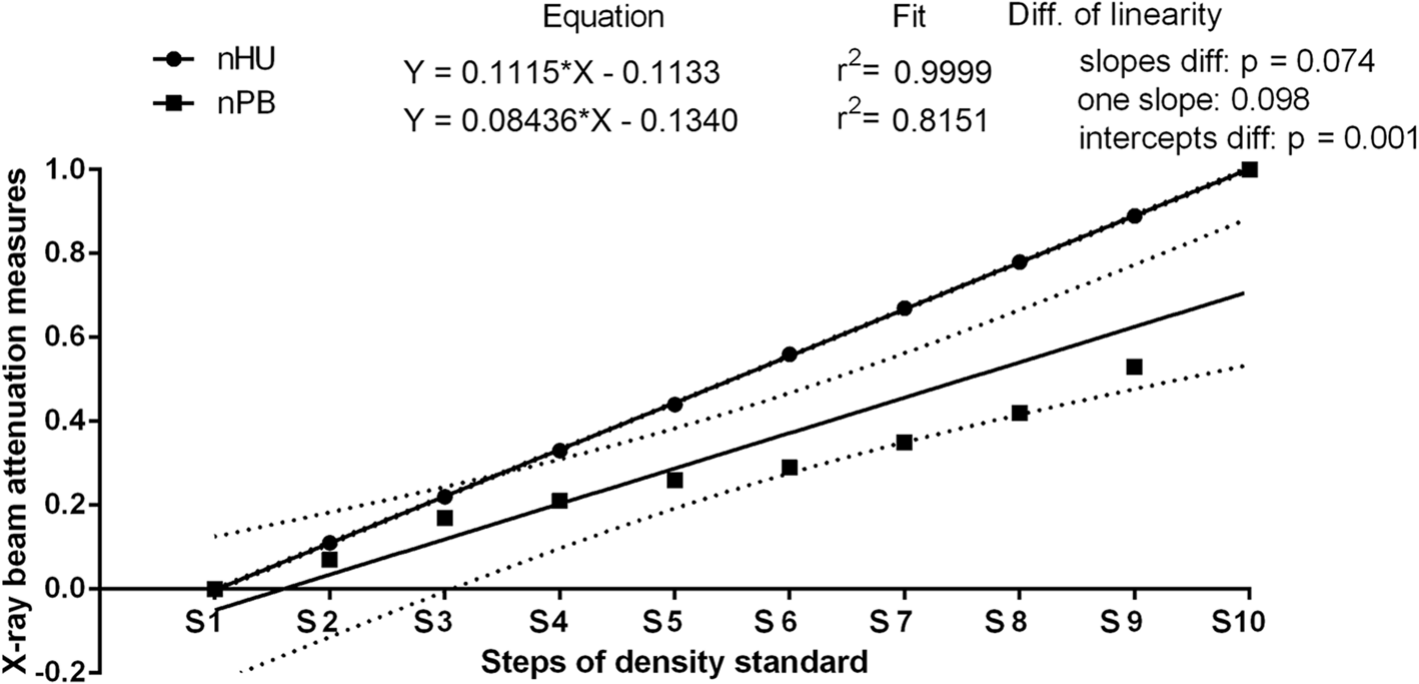
Comparison of normalized Hounsfield unit (nHU) and normalized pixel brightness (nPB) throughout ten steps of density standard (S1–S10). Similarity was tested using linear regressions and considered significant for *p* < 0.05. If the difference between slopes was not significant (*p* > 0.05), a single slope measurement was calculated

The similarities between nHU and nPB were tested using the linear regression model. The slope of the linear regression equations for nHU compared to the slopes of nPB were not significantly different (*p* = 0.074), and a single slope measurement was calculated (one slope = 0.098; Fig. 6). The intercept within an nHU and nPB data pair was compared and considered significant (*p* = 0.001), thus, one intercept was not calculated. Furthermore, similarity between these two X–ray beam attenuation measures can be observed.

As a result of the EOTRH group classification of the horses' incisor teeth, 105 incisor teeth passed the criteria of grade–related EOTRH group 0, 195 incisor teeth passed the criteria of grade–related EOTRH group 1, 111 incisor teeth passed the criteria of grade–related EOTRH group 2, and 61 incisor teeth passed the criteria of grade–related EOTRH group 3. On this basis the structure of maxillary incisor teeth classification was as follows: grade 0 (normal teeth) *n* = 105, grade 1 (mild EOTRH) *n* = 195, grade 2 (moderate EOTRH) *n* = 111, and grade 3 (severe EOTRH) *n* = 61. In total, eight incisor teeth were excluded from the study due to clinical and radiographic signs of the teeth encompassing the following problems: loose teeth (*n* = 2), transverse fractures (*n* = 3), sagittal fractures (*n* = 2), and infundibular caries (*n* = 1), consequently the total number of 472 incisor teeth were further investigated.

Comparing NP values between the S1–S10 for each EOTRH–related group separately, the NP values gradually increased, from the lowest values in S1 to the highest values in S10, regardless of the EOTRH grade (Fig. 3). This increase in the extracted image brightness indicator was significant from S6 in EOTRH 0 (*p* < 0.0001; Fig. 3A), from S3 in EOTRH 1 (*p* < 0.0001; Fig. 3B), from S5 in EOTRH 2 (*p* < 0.0001; Fig. 3C), and from S6 in EOTRH 3 (*p* < 0.0001; Fig. 3D). Moreover, in EOTRH group 0, one may observe the differences in NP values between S1–S4 and S8–S10, S5–S7 and S8–S10, S7–S8 and S9–S10, as well as S9 and S10. In EOTRH group 1, the most differences were noted in NP values, namely between S3–S4 and S5–S10, S5–S7 and S8–S10, S7–S8 and S9–S10, as well as S9–S10. In EOTRH group 2, the differences in NP values were observed between S4–S7 and S8–S10, S6–S8 and S9–S10, as well as S9–S10. Similarly in EOTRH group 3, the differences in NP values were noted between S4–S8 and S9–S10 as well as S8–S10.

**Fig. 7:**
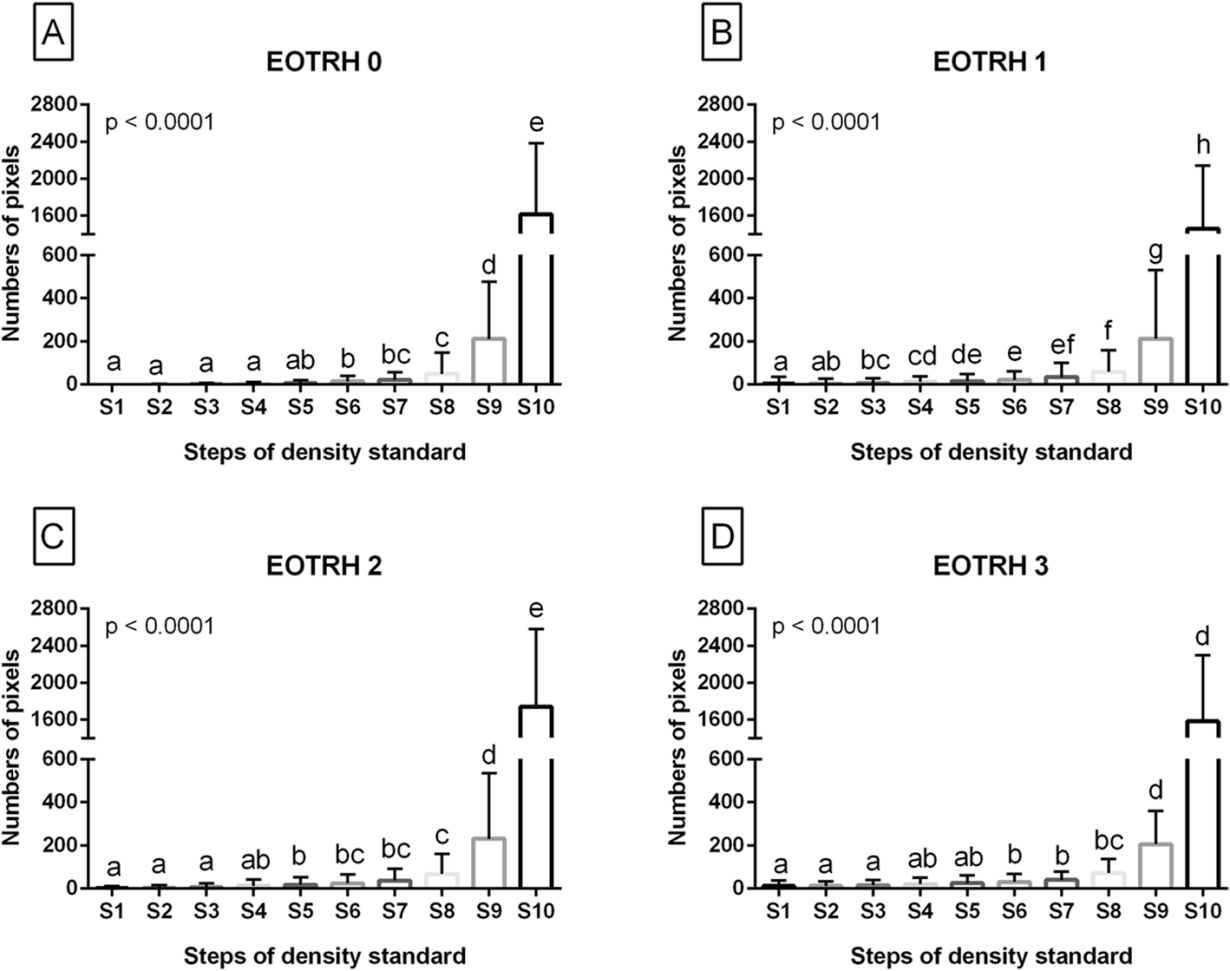
The comparison of the numbers of pixels (NP) between Equine Odontoclastic Tooth Resorption and Hypercementosis (EOTRH) grades (EOTRH 0–3). Data displayed separately for consecutive steps of density standard—S1 (A), S2 (B), S3 (C), S4 (D), S5 (E), S6 (F), S7 (G), S8 (H), S9 (I) and S10 (J). Lower case letters (a–c) indicate differences between groups for *p* < 0.05. The significant increase with the EOTRH grades is marked with colored lines – orange line when the increase was noted between EOTRH 0 and 3, red line when the increase was observed between EOTRH 1 and 3, blue line when the increase was observed between EOTRH 0 and 1, and green line when the increase occurred between EOTRH 2 and 3

Comparing NP values between the EOTRH grades for each S–labelled data series separately, NP values increased from the lowest values in EOTRH 0 to the highest in EOTRH 3 in S1–S8 (Fig. 7A–H) but not S9–S10 (Fig. 7I–J). One may observe two patterns of the increase in the extracted image brightness indicator. In the first pattern, the differences in NP values were noted between EOTRH 0 and 3 as well as EOTRH 1 and 3, and marked by orange and red lines, respectively. Whereas in the second pattern, the differences in NP values were observed between EOTRH 0 and 3, EOTRH 0 and 1, as well as EOTRH 2 and 3, and marked by orange, blue, and green lines, respectively. The first pattern was recognized for S1 (*p* < 0.0001; Fig. 7A), S2 (*p* < 0.0001; Fig. 7B), S6 (*p* < 0.0001; Fig. 7F), S7 (*p* < 0.0001; Fig. 7G), and S8 (*p* < 0.0001; Fig. 7H); whereas the second one for S3 (*p* < 0.0001; Fig. 7C), S4 (*p* < 0.0001; Fig. 7D), and S5 (*p* < 0.0001; Fig. 7E).

For the shown differences in NP values between the EOTRH grades, the accuracy of the differentiation of EOTRH 0 and 3, EOTRH 1 and 3, EOTRH 0 and 1, as well as EOTRH 2 and 3 was calculated, respectively (Table 2). For the first threshold (mean), the highest Se (0.38) was noted for EOTRH 0 and 3 in S6, EOTRH 1 and 3 in S6, EOTRH 0 and 3 in S7, as well as EOTRH 1 and 3 in S7. Se ranged from referred 0.38 to 0.17 for EOTRH 0 and 1 in S7; whereas Sp ranged from 1.00 for EOTRH 0 and 3 in S1 to 0.74 for EOTRH 1 and 3 in S7. For the second threshold (|mean – SD|), the highest Se (0.98) was observed for EOTRH 0 and 3 in S8 as well as EOTRH 1 and 3 in S8. Se ranged from referred 0.98 to 0.08 for EOTRH 0 and 1 in S3; whereas Sp ranged from 1.00 for EOTRH 0 and 3 in S1 to 0.31 for EOTRH 1 and 3 in S8. One may observe that the Se of differentiation of EOTRH 0 and 1 increased in the case of both thresholds used, from the lowest in S3, higher in S4, to the highest in S5, despite that for each of the awarded steps low Se and high Sp was noted.

**Table 2:** The accuracy of the differentiation of radiographic signs of selected EOTRH 0 – 3 grades based on the numbers of pixels (NP) of the selected pixel brightness (PB) in consecutive steps of density standard (S1–S10). Sensitivity (Se), specificity (Sp), positive predictive value (PPV), and, negative predictive value (NPV) were calculated using two thresholds (mean and |mean – SD|)

Step	EOTRH		Se	Sp	PPV	NPV	Se	Sp	PPV	NPV
	lower	higher	mean				|mean – SD|			
S1	0	3	0.25	1.00	1.00	0.70	0.25	1.00	1.00	0.70
S2	0	3	0.30	0.99	0.95	0.71	0.30	0.96	0.82	0.70
S3	0	3	0.31	0.96	0.83	0.71	0.41	0.96	0.86	0.74
S4	0	3	0.36	0.95	0.81	0.72	0.49	0.89	0.71	0.75
S5	0	3	0.23	0.93	0.67	0.68	0.52	0.74	0.54	0.73
S6	0	3	0.38	0.82	0.55	0.69	0.67	0.68	0.55	0.78
S7	0	3	0.38	0.79	0.51	0.69	0.97	0.40	0.48	0.95
S8	0	3	0.34	0.78	0.48	0.67	0.98	0.46	0.51	0.98
S1	1	3	0.25	0.96	0.65	0.80	0.25	0.96	0.65	0.80
S2	1	3	0.30	0.94	0.60	0.81	0.30	0.91	0.51	0.81
S6	1	3	0.38	0.79	0.36	0.80	0.67	0.57	0.33	0.85
S7	1	3	0.38	0.74	0.32	0.79	0.97	0.32	0.31	0.97
S8	1	3	0.34	0.76	0.31	0.79	0.98	0.31	0.31	0.98
S3	2	3	0.30	0.90	0.65	0.67	0.39	0.83	0.59	0.68
S4	2	3	0.34	0.85	0.59	0.67	0.46	0.74	0.53	0.68
S5	2	3	0.33	0.80	0.51	0.65	0.33	0.75	0.46	0.64
S3	0	1	0.17	0.94	0.85	0.38	0.08	0.96	0.79	0.36
S4	0	1	0.21	0.91	0.82	0.38	0.16	0.94	0.84	0.38
S5	0	1	0.25	0.80	0.70	0.36	0.23	0.82	0.70	0.36

## Discussion

The techniques to enhance early detection of radiographic signs of dental diseases in horses is an important direction of recent research in equine dentistry [2, 38, 52–54]. Most recently, filtering algorithms and texture analysis of incisor teeth radiographs, based on the first– and second–order statistics [38] and two–dimensional entropy measures [52], have been successfully used in EOTRH 0 and 3 differentiation. Visually, it is less difficult to identify radiographic signs of EOTRH 3, representing severe tooth hypercementosis and resorption, and EOTRH 0, representing healthy teeth [32]. Therefore, one may conclude the use of digital processing techniques in these cases will enhance the quality of the maxillary incisor radiographs so that the radiologist can more easily identify the radiographic signs. However, no clear differentiation of radiographic signs of EOTRH 1, representing mild tooth hypercementosis and resorption [32], and EOTRH 0, were evidenced [38, 52]. In the case of visual inspection, early signs of EOTRH might be missed by merely personal evaluation of radiographs. Therefore, specific detection of early radiographic signs of EOTRH is a major component of using digital processing of radiographs to enhance automated disease detection. The first step in achieving this goal using the density standard and scaled–pixel–counting protocol was presented in the current preliminary research.

**Fig. 8:**
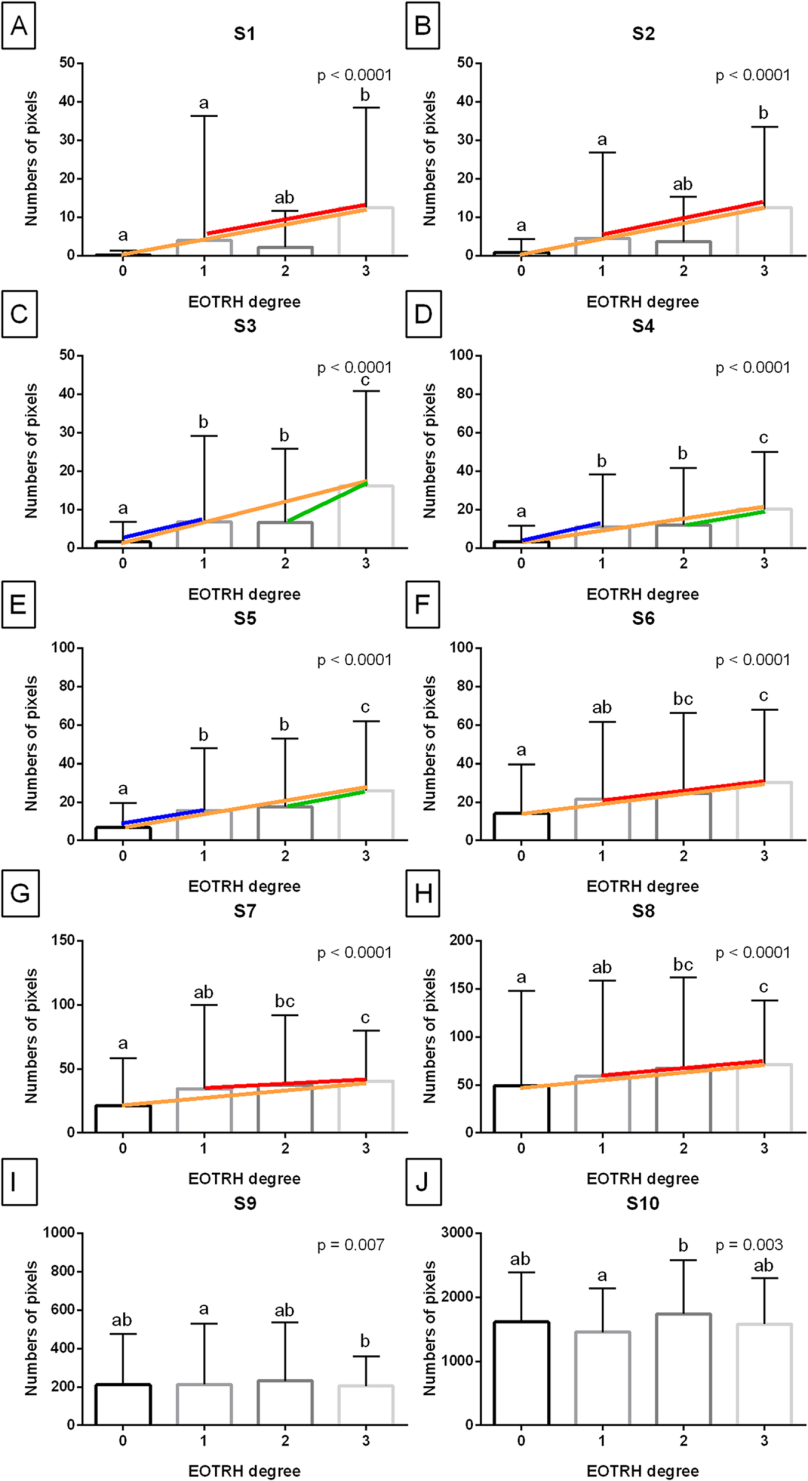
The comparison of the numbers of pixels (NP) between ten steps of density standard (S1-S10). Data displayed separately for consecutive Equine Odontoclastic Tooth Resorption and Hypercementosis (EOTRH) grades – EOTRH 0 (A), EOTRH 1 (B), EOTRH 2 (C), and EOTRH 3 (D). Lower case letters (a–h) indicate differences between groups for *p* < 0.05

The demonstration of the strong positive correlation between mean values of HU and PB points to the relationship between a linear transformation of the original linear X–ray beam attenuation coefficient measurement into the HU scale [11] and a linear transformation of the pixel brightness degree measurement into the PB scale. This linear relationship was confirmed by linear regression equations for nHU and nPB indicating the similarity between these two X–ray beam attenuation measures, the classic direct (nHU) [11, 36] and the new indirect (nPB) one. As the X–ray beam attenuation increases with tissue thickness [55] the experimentally determined linearity was confirmed in a clinical study by an increase in the NP value with an increase in the number of steps of density standard. This increase can be seen in Fig. 8 irrespective of EOTRH grades. These observations justify the use of density standard for indirect quantification of the brightness of the radiograph, and thus the use of the proposed scaled–pixel–counting protocol for indirect quantification of the radiographic signs of tooth resorption and hypercementosis on the EOTRH syndrome model. Recent research has discussed the use of histogram–based and matrix–based texture features [38] and two–dimensional entropy measures [52] in the digital processing of EOTRH radiographs. These studies were focused only on maxillary, not maxillary and mandibular, incisor teeth. The maxillary incisor teeth were considered as the best choice for this preliminary research due to the lowest superimposition of surrounding tissues compared to other horse teeth [45]. Therefore, the implementation of the scaled–pixel–counting protocol to quantify and compare the image structure of teeth and density standard was focused similarly, only on teeth 101, 102, 103, 201, 202, and 203. Apart from the computational part, the only modification to the standard equine dental radiographs acquisition concerned the attachment of the density standard to the radiographic cassette. Thus, use radiography according to the protocol adapted in the current study is no more contraindicated than a complete protocol of the dental examination. In many cases of equine dental diseases, a visual examination of the oral cavity allows the veterinarian to identify and manage dental problems and diagnose diseases in their early stages [45]. In some diseases, such as EOTRH [32, 56], teeth fractures [1, 57, 58], teeth infections [1, 57], or remodelling and lysis of alveolar bone [1, 57, 59], dental radiographic imaging is beneficial. This advantage can be seen in Fig. 6, where the signs of successive EOTRH grades are radiographically, rather than visually, visible.

In the current study, the differences in NP values were noted between EOTRH 0 and 3, EOTRH 0 and 2, as well as EOTRH 0 and 1. The EOTRH groups 0 and 3 are easily differentiated visually based on the radiographic signs. In the current study, most of the teeth in EOTRH group 3 showed a smooth outline despite the obvious internal changes, whereas, on recent raw [32, 37, 56, 60] and digitally processed [38, 52] radiographs, the signs of obviously irregular or rough incisor tooth surfaces were predominately. In recently published research, the accuracy of EOTRH group 0 and 3 differentiation ranged from 0.25 Sp and 0.99 Se for matrix–based texture features to 0.50 Sp and 1.00 Se for two–dimensional entropy measures [52]. In the current study, the accuracy of EOTRH 0 and 3 differentiation ranged from 0.25 Sp and 1.00 Se for S1 to 0.38 Sp and 0.82 Se for S6. In both cases, the mean threshold was used and the achieved results were at a similar level. However, the accuracy of NP–depended differentiation of referred EOTRH grades increased when |mean–SD| threshold was applied. Thus, one may conclude, an appropriate selection of threshold may improve the effectiveness of detecting radiographic signs of the disease, however further research is required. Noteworthy in the current study is that the accuracy of EOTRH 0 and 1 differentiation reached the level of 0.25 Sp and 0.80 Se, which indicates that the used indirect assessment of radiodensity of equine incisor teeth makes it possible to differentiate early radiographic signs of mild EOTRH [32] from healthy incisor teeth. Although the sensitivity to distinguish between these two is not sufficiently high, the achieved results justify the need for further research on the use of the density standard and scaled–pixel–counting protocol in digital processing of radiographs in order to provide automated disease detection. The current study was based on the raw radiographs collected directly from the X–ray scanner, which were not digitally processed, therefore the use of radiograph filtering may enhance the quality of the radiographs and thus, increase the Sp and Se of detecting early radiographic signs of EOTRH. Filtering, which enhances brightness, contrast, and/or edges of radiographs, is routinely used in human dentistry [61, 62] and has been introduced into equine dentistry [38, 52], and this direction of further research development seems to be promising.

## Conclusion

The scaled–pixel–counting protocol based on the use of density standard has been successfully implemented for the differentiation of radiographic signs of EOTRH degrees. The linear relationship between an original X–ray beam attenuation coefficient measured in the HU scale and a new pixel brightness degree measured in the PB scale was confirmed, thus the NP values representing incisor teeth structure and consecutive steps of density standard were compared. The NP values increase with the number of steps of density standard as well as with EOTRH degrees, confirming the clinical usefulness of the PB–counting model in assisted differentiation of radiographic signs of EOTRH grades. Noteworthy, similar accuracy of the EOTRH grade differentiation was noted for data pairs EOTRH 0–3 and EOTRH 0–1, one may suggest the presented protocol may hereafter be applied to automated detection of both late and early radiographic signs of tooth resorption and hypercementosis.

## Data Availability

All data generated or analyzed during this study are included in this published article. If any additional material used and/or analyzed during the current study is required, these are available from the corresponding author on reasonable request.

## Abbreviations

AREAs: Regions of interest representing S1–S10
CBCT: Cone–beam computed tomography
CT: Computed tomography
EOTRH: Equine Odontoclastic Tooth Resorption and Hypercementosis
FBCT: Fan–beam computed tomography
hr MRI: High resolution–magnetic resonance imaging
hr pQCT: High–resolution peripheral quantitative computed tomography
HU: Hounsfield units
MDCT: Multi–detector computed tomography
MIMICS: Materialises interactive medical image control system
nHU: Normalized Hounsfield unit
NP: Number of pixels
nPB: Normalized pixel brightness
NPV: Negative predictive value
PB: Pixel brightness
PPV: Positive predictive value
r: The Pearson correlation coefficient
ROI: Region of interest
S1–S10: Ten steps of the density standard
SD: Standard deviation
Se: Sensitivity
SEM: Scanning electron microscope
Sp: Specificity
